# Effect of male mating history and age on remating by female European corn borer

**DOI:** 10.1371/journal.pone.0175512

**Published:** 2017-04-06

**Authors:** Panagiotis G. Milonas, George K. Partsinevelos, David A. Andow

**Affiliations:** 1 Benaki Phytopathological Institute, Department of Entomology and Agricultural Zoology, Kifisia, Greece; 2 Department of Entomology, University of Minnesota, Saint Paul, Minnesota, United States of America; CNRS, FRANCE

## Abstract

If mating with an inferior male has high fitness costs, females may try to avoid mating with these males. Alternatively, females may accept an inferior male to ensure they have obtained at least one mate, and/or to avoid the costs of resisting these males. We hypothesized that females compensate for mating with an inferior male by remating. We tested this hypothesis by measuring remating propensity in females that had mated with an old, multiply-mated male, a 9-day-old virgin male, or a young, virgin male. Females were more likely to remate when they had mated with multiply-mated males than when they had mated with a 9-day-old or young virgin male. We discuss the observed mating behavior by females in terms of sexual selection for multiple mating.

## Introduction

Although one mating is often sufficient for females to maximize their reproductive effort, females across many different animal taxa mate multiply [1]. Females may gain considerable benefits from multiple mating. These include direct benefits such as nutrients, parental care, higher quality breeding areas, protection, and other resources and conditions provided by males that result in higher female fitness [2–6]. Females may also derive genetic benefits from remating [7], such as decreased vulnerability to harmful genetic elements [8, 9] or reduced risk of genetic incompatibilities [10]. Furthermore, females may mate multiply to improve on previous matings with a genetically inferior male [11]. This is most likely to occur when access to preferable males is limited or a delay in mating has negative effects in reproduction [11].

For males, multiple mating can provide a higher lifetime fitness by siring more offspring from multiple females. Females, on the other hand, can maximize their fitness with a limited number of matings [12]. These divergent selection pressures for males and females inevitably result in sexual conflict [13]. Males are selected to develop mechanisms to seduce or coerce females to mate beyond what would be their optimum rate [14, 15]. Mating for females might be costly due to energy loss, increased mortality and changes in female physiology [1, 16], and consequently, females might evolve defense mechanisms to discriminate male quality and avoid persistently courting, low-quality males [14, 17, 18].

Kokko and Mappes [19] argued that the question should not be ‘why should a female multiply mate?’ but ‘why should a female not mate in all mate encounters?’ This perspective may be most acute when mate encounters are rare and highly unpredictable so that comparisons of potential mates are difficult or impossible [20]. After all, not mating at all is by far more costly for a female than mating with an inferior male [19]. These theoretical concepts suggest that females may modify their propensity to re-mate depending on the quality of the previous and current male.

Here, we experimentally evaluated the influence of previous male mating history on the remating behavior of females and its consequences for lifetime fitness in European corn borer, *Ostrinia nubilalis* (Hübner), Lepidoptera: Crambidae. Female *O*. *nubilalis* suffer a significant cost in total reproduction and lifespan when mating with old virgin males or multiply-mated males in comparison to mating with a young virgin male [21, 22]. In the present study, we determined if females that had mated previously with a multiply-mated male or an old virgin male would show a greater propensity to mate with a young virgin male, than when they had mated with a young, virgin male. Further, we considered if this behavior allows females to compensate fitness for previously mating with an old or multiply-mated male.

## Materials and methods

### Insects

*Ostrinia nubilalis* pupae were collected from infested maize fields in Northern Greece and transported to Benaki Phytopathological Institute insectaries until adults emerged. Nondiapausing larvae were reared at 25±1°C, 70±10% RH and a photoperiod of 16:8 L:D. Adults were maintained in the laboratory in a 16:8 L:D photoperiod, at 27°C in daylight and at18°C in dark and ca. 80% relative humidity [23]. All pupae were sexed prior to eclosion [24] to ensure isolation, and placed into separate cages until adult emergence. Newly emerged adults were transferred to the colony or to the experimental cages.

### Experimental design

To determine if a prior mating experience influenced the probability of remating, we set up an experiment providing females with one of three mating experiences that were known from previous research to differentially affect female fecundity and longevity: multiply-mated males, old virgin males the same age as the multiply-mated males, and young virgin males. Fecundity and longevity was highest when females mated with young virgin males and lowest when they mated with multiply-mated males [21, 22]. Females mated with similar frequency when provided with either young and old virgin males [21], and multiply-mated males secured more mates than young virgin males, probably because they were more persistent in mating attempts [22]. Thus, if the probability of remating is higher after a low quality mating, we hypothesize that remating will be highest after mating with a multiply-mated male and lowest after mating with a young virgin male.

#### Multiply mated males

Males with multiple matings were obtained as described by [22]. We used 150 males to obtain 88 multiply mated males. These males were paired with a newly emerged virgin female and observed during the scotophase to note mating. After mating, the multiply mated males were removed and female longevity and fecundity was measured. These mated females were randomly sorted into two groups: one group (61 females) was allowed to remate as described below, and the other group (27 females) was not allowed to remate.

#### Old virgin males

Emerged adult males were isolated in plastic cages 12cm diameter x 6cm height with a net lid cover. In each cage no more than 10 males were placed. When males reached their 9^th^ day after emergence they were used in the experiment. We used this age class as we have shown previously that they confer lower direct benefits to females than young virgin males [21] and they were the same age as most of the multiply mated males. One virgin 9-day-old male was placed with a young newly emerged virgin female in a mating cage and observed during the scotophase to note mating. We formed 132 pairs of virgin females with old virgin males to obtain 86 females that had mated with an old virgin male. These females were allowed to remate and longevity and fecundity were measured as indicated below.

#### Young virgin males

Young virgin males were used the day they emerged. We formed 40 pairs of young virgin males and females in a mating cage and observed during the scotophase to note that mating occurred in 33 pairs. These females were allowed to remate and longevity and fecundity were measured as indicated below.

#### Remating

For remating, females were provided a new virgin male 2–3 days old. All pairs were observed during the subsequent scotophases for mating. Males were replaced with a new virgin male every 2 days or after a mating. The day of each remating for females was recorded until her death. In each mating cage adult food and an oviposition substrate (wax paper on sides of the cage) were renewed every day to measure daily fecundity by females for their total lifetime.

### Statistical analysis

The probability of remating among female groups was analyzed by performing log-likelihood analysis on our data on females that remated versus those that did not remate and on the number of rematings for each female, with Bonferroni correction for significance levels. Time to remate was analyzed using parametric survival analysis [25]. We assumed that remating events might become more frequent with ageing females, and used a Weibull hazard function *h = ρt*^*κ*^, where *t* is the age of a live female, *ρ* is the initial remating rate at age 0, and *κ* is the rate of increase or decrease in the remating rate [25]. If *κ* is >1 (or <1), then remating rate increases (or decreases) with age and if *κ* = 1, then remating rate is constant with age. We used maximum likelihood using both censored and non-censored data in Mathematica version 5.2 (Wolfram Research, Inc., Champaign, IL, U.S.A.) to estimate all parameters of these equations following Cox and Oakes (25).

Longevity data of females with different mating histories was also analyzed with parametric survival analysis with a Weibull hazard function, comparing remated and non-remated females in each of the three mating histories. In this analysis, *t* is the age of a live female, *ρ* is the initial mortality rate at age 0, and *κ* is the rate of increase or decrease in the mortality rate with age. In addition, we tested for a significant relationship between lifetime fecundity and female longevity between females with different mating histories by conducting an analysis of covariance (ANCOVA). We tested for homogeneity of slopes (lifetime fecundity) for each group of females with average lifetime fecundity as the response variable, female longevity as the covariate, and female mating history as the independent factor. A significant covariate by factor interaction is evidence that the relationship between longevity and fecundity varied among females with different mating history. For females that mated with multiply-mated males, these analyses compared females that remated with those that had no opportunity to remate. For the other two groups, the analyses compared those that remated with those that did not remate. Generalized linear mixed-effect models with Poisson error were used to analyze average total and daily fecundities in relation to the mating history. Pairwise comparisons were performed with Sidak test with significance at a = 0.05. All analysis was done with SPSS (SPSS 21).

## Results

### Remating

**Fig 1:**
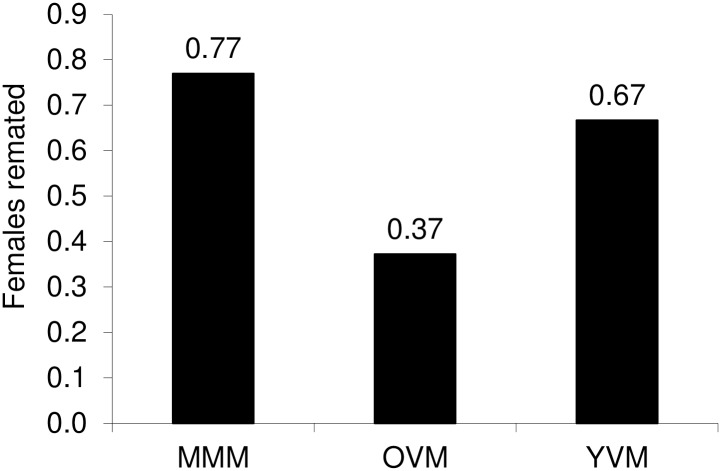
Probability of *Ostrinia nubilalis* females remating after first mating with a young virgin male (YVM), an old virgin male (OVM), or a multiple mated male (MMM).

The probability that females would remate was affected by the state of the first male partner (*χ*^2^ = 23.64; df = 2; *P* = 1.16x10^-6^) (Fig 1). Females that had mated first with a multiply-mated or young virgin male were about twice as likely to mate again than those mated for the first time with an old virgin male (*χ*^2^ = 22.58; df = 1; *P* = 2.01x10^-6^), but the remating probability was similar in these two groups (*χ*^2^ = 1.06; df = 1; *P* = 0.303). Females that mated with a multiply-mated male remated significantly more often than females that mated with either old or young virgin males (*χ*^2^ = 10.40; df = 2; *P* = 0.006). After mating with a multiply-mated male, 27% of females mated a third time and 10% of females mated a fourth time, with an average of 1.84 rematings. None of the females mated more than twice when first mating with either a young or old virgin male.

**Fig 2:**
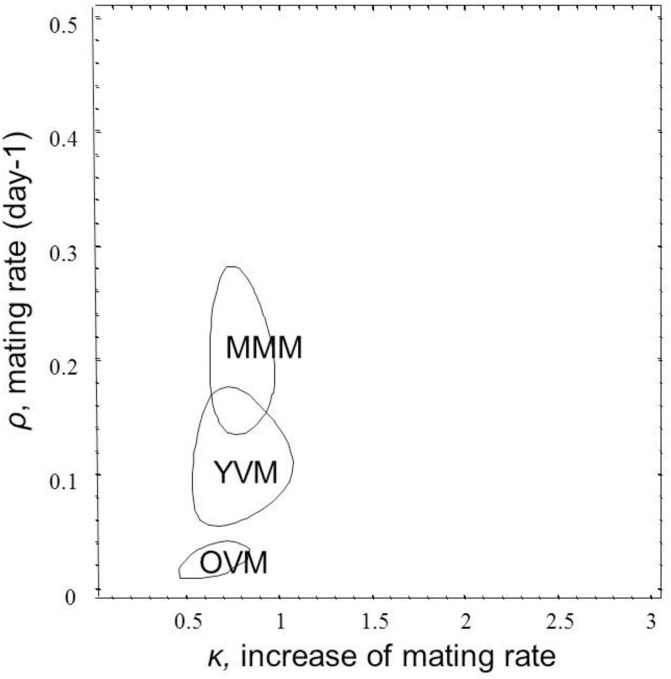
Fitted parameters for Weibull hazard function for the time to remating for *Ostrinia nubilalis* females that mated first with a young virgin male (YVM), an old (9 days old) virgin male (OVM) or a multiple mated male (MMM). The lines enclose 95% confidence regions for the joint parameter estimates.

The period of time to remating was significantly different among the three groups (Fig 2). Females that mated with a multiply-mated male had their next mating on average in 1.70 days (N = 47). Those that mated with a young virgin male had their second mating in 2.62 days (N = 22) and those that mated with an old virgin male had their second mating in 3.42 days (N = 31). Parametric survival analysis with a Weibull hazard function revealed that the initial rate of remating (*ρ*) was lower after mating with an old virgin male and faster after mating with a multiply mated male. In addition, κ<1 for all of the mating groups, showing that it was less likely for females to remate as they aged (Fig 2).

### Effects of remating

**Fig 3:**
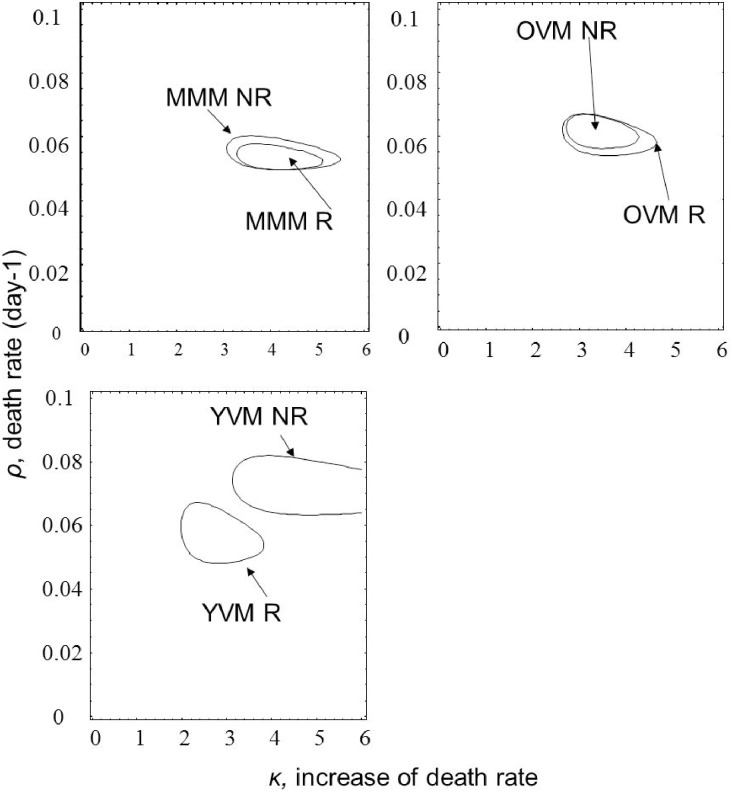
Fitted parameters for Weibull mortality function for *Ostrinia nubilalis* females that mated first 1) with a young virgin male and didn’t remate (YVM-NR) or did remate (YVM-R), 2) with an old (9 days old) virgin male and didn’t remate (OVM-NR) or did remate (OVM-R) and 3) with a multiple mated male and didn’t remate (MMM-NR) or did remate (MMM-R). The lines enclose 95% confidence regions for the joint parameter estimates.

Parametric survival analysis revealed that remating had a positive influence on female’s longevity only for females that mated initially with a young virgin male, because the confidence regions for the two groups did not overlap (Fig 3). For these females, the initial mortality rate, *ρ*, was significantly higher for females that did not remate than for females that remated (Fig 3). As the females that did not remate also had an initial mortality rate, *ρ*, significantly higher than all other groups, except for females that did not remate after mating with a multiply mated male, it is possible that they were lower quality females than in the other groups. In all cases, females had increasing mortality rates as they aged no matter which male type they had mated with first, or if they remated or not (Fig 3, κ>1 for all cases).

**Fig 4:**
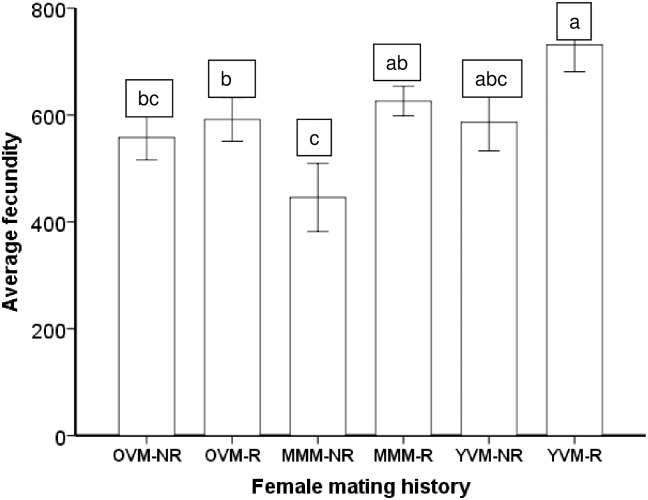
Average fecundity for *Ostrinia nubilalis* females that mated first with a multiple mated male (MMM), an old (9 days old) virgin male (OVM) or a young virgin male (YVM), and then remated (R) or not (NR). Means with different letters are significantly different (Sidak Test a = 0.05)

**Fig 5:**
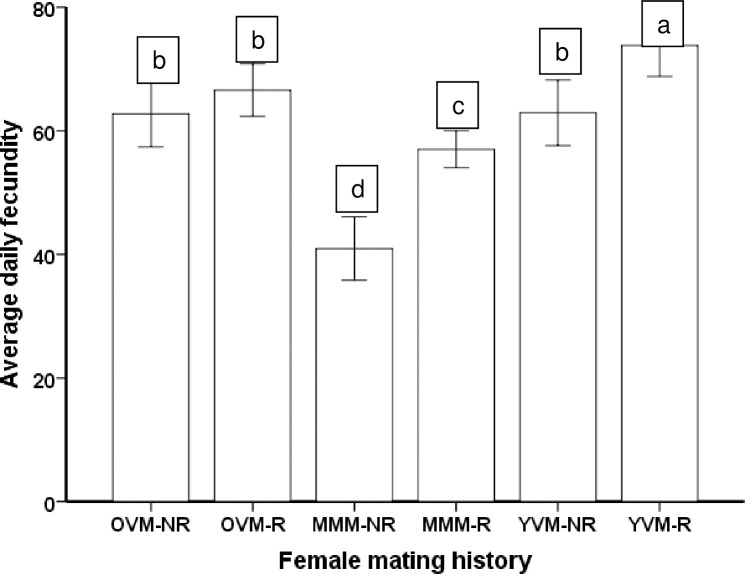
Average daily fecundity (± SE) for *Ostrinia nubilalis* females that mated first with a multiple mated male (MMM), an old (9 days old) virgin male (OVM) or a young virgin male (YVM), and then remated (R) or not (NR). Means with different letters are significantly different (Sidak Test, a = 0.05)

Among females that first mated with multiply-mated males, females that remated had higher fecundities than the ones that had no opportunity to remate (Fig 4). However, among females that first mated with old virgin males or young virgin males, remating had no influence on their fecundity (Wald *χ*^2^ = 1608; df = 5; *P*<0.0001) (Fig 4). Similarly, average daily fecundities varied with female mating history (Wald χ^2^ = 252.2; df = 5; P<0.0001). Females that mated first with a young virgin male and mated again had the highest daily fecundity (Fig 5). In addition, we found no evidence for a trade-off between fecundity and longevity associated with female mating history. The slopes of fecundity versus longevity were not different among or within any of the female mating histories (interaction effect, F_5_, _146_ = 1.28; P = 0.276), although, as expected, average fecundity increased with increasing female longevity (main effect, F_1_,_146_ = 29.37; P<0.0001).

## Discussion

Previous work has shown that females mating with multiply-mated *O*. *nubilalis* males suffered reduced fitness compared to mating with equal-aged or young virgin males [22]. Multiply-mated males secured more mates when in competition with same-aged virgin males [22, 26] mainly because of their higher persistence in courtship behavior [22]. Although females discriminated against multiply-mated males, their resistance was not strong enough to avoid mating with these males [22]. In *O*. *nubilalis*, it is plausible that females avoid the costs of resisting mating with multiply-mated males and make up the fitness deficit by mating with multiple males. A delay in mating for *O*. *nubilalis* females may have considerable detrimental impact on their lifetime fitness [27]. Consequently, females should be eager to mate soon after emergence and may limit their resistance to low quality mates to avoid the costs of delayed mating or not mating at all [19].

Our results here showed that the mating history of the previous mate influenced female remating behavior. Females that mated with multiply-mated males remated sooner (1.70 versus 2.62 days) and more often (1.84 versus 1.0 times) than the females that first mated with young virgin males, even though the cumulative probability of remating was similar. This suggests that females can recognize when they have mated with a multiply-mated male, and change their mating behavior. During copulation males form a spermatophore that is transferred and stored in the bursa copulatrix of the females [28]. The size of the spermatophore and the degree that it fills the bursa copulatrix are cues a female can use to recognize poorer quality mates [29]. In *O*. *nubilalis* [22, 30], as is true for many Lepidoptera, male ejaculates are smaller with repeated mating, so the spermatophores of multiply-mated males are smaller than of virgin males.

The effect of mating with a multiply-mated male carried over to affect the probability of remating a second and third time. Perhaps females that have received a small amount of ejaculate (nutrient or sperm) in their first mating remate to acquire additional ejaculate. In contrast, no female that mated first with a young or old virgin male remated more than once, suggesting that the carry-over effect from the first mating with a multiply-mated male was substantial. It is possible that virgin males mating with virgin females transferred a large spermatophore, while virgin males mating with an already mated female transferred a smaller one that did not fill the bursa copulatrix [31, 32]. An alternative explanation for the carry-over effect is that males were strategically tailoring their ejaculate contents. In a closely related pyralid moth (*Plodia interpunctella*), males transferred an ejaculate rich in nonfunctional apyrene sperm when mating with a virgin female to delay female remating (defensive ejaculate function). Yet, when mating with an already mated young female, males decreased the proportion of nonfunctional sperm in their ejaculate and transferred mostly eupyrene sperm to ensure paternity (offensive ejaculate function) [33]. Hence, it is possible that multiply-mated males had little apyrene sperm to transfer, so their mates were more eager to remate, while the second young virgin male transferred little apyrene sperm due to female mating history, resulting in continued high female remating propensity.

In addition, the remating increased the lifetime and daily fitness of females, nearly restoring it to levels equivalent to mating once with a young virgin male. Thus, females may avoid resisting mating with low-quality multiply-mated males, and compensate for the fitness loss by increasing the rate and frequency of remating.

In a previous study we have found that females suffered reduced fitness when mating with old virgin males compared to young virgin males, and did not discriminate against mating with older virgin males [21]. Here we found that females had a lower cumulative probability of remating, and remated later when they first mated with older virgin males than with younger virgin males. Old virgin males produced a larger spermatophore than young virgin males [21], so old virgin males may be masking their “inferior quality” to females [34, 35] and thereby ensure paternity before they die. Overall, in the present study remating seemingly increased female fitness after mating with an old virgin male.

Finally, we found that remating increased female fitness even when she first mated with a high quality, young virgin male, although this could have been because females choosing not to remate were of lower quality than those that did remate. Overall, our study suggests that female *O*. *nubilalis* should be under positive selection pressure for polyandry or remating, regardless of the identity of the first mate. In field conditions however, females are usually single-mated and the highest observed ratio of twice mated females was just 40% [36], which would make this species a mildly polyandrous species [37]. Since polyandry seems to be beneficial, why it is not more common? We cannot exclude the possibilities that lack of mating opportunities could be a reason for low mating frequencies in the wild or that polyandry is only beneficial under some unknown conditions. However, the answer could lie within the antagonistic sexual coevolution between males and females [17]. Males may be interested in reducing subsequent female matings [38], and large spermatophore size may be a mechanism to ensure paternity by reducing female remating rate or increasing remating intervals [39]. It is also possible that female monandry is imposed by males through transfer of a chemical substance or physical blockage that reduces the likelihood of female remating [40, 41]. Chemical substances or the amount of viable sperm after mating may make females cease pheromone production and eventually prolong their refractory period. The cessation of pheromone production in females, known as pheromonostasis, has been found to be directly related to male quality and the amount of viable sperm transferred during copulation [42, 43]. In any event, the reasons for mild polyandry in this species remain to be understood.

Overall our results supported our hypothesis that female *O*. *nubilalis* avoid the costs of resisting mating with multiply-mated males and make up the fitness deficit by remating with multiple males [22].
